# Repeat or single-dose lentiviral vector administration to mouse lungs? It’s all about the timing

**DOI:** 10.1038/s41434-023-00403-3

**Published:** 2023-05-10

**Authors:** Martin Donnelley, Patricia Cmielewski, Emma Knight, Chantelle Carpentieri, Alexandra McCarron, Nathan Rout-Pitt, David Parsons, Nigel Farrow

**Affiliations:** 1 Respiratory and Sleep Medicine, Women’s and Children’s Hospital, Adelaide, SA Australia; 2 Adelaide Medical School, The University of Adelaide, Adelaide, SA Australia; 3 Robinson Research Institute, The University of Adelaide, Adelaide, SA Australia; 4 South Australian Health and Medical Research Institute, Adelaide, SA Australia; 5 School of Public Health, University of Adelaide, Adelaide, SA Australia; 6 College of Medicine, University of Vermont, Burlington, VT USA

**Keywords:** Respiratory tract diseases, Gene therapy

## Abstract

Lentiviral vectors are attractive delivery vehicles for cystic fibrosis gene therapy owing to their low immunogenicity and ability to integrate into the host cell genome, thereby producing long-term, stable gene expression. Nonetheless, repeat dosing may be required to increase initial expression levels, and/or boost levels when they wane. The primary aim of this study was to determine if repeat dosing of a VSV-G pseudotyped LV vector delivered into mouse lungs is more effective than a single dose. C57Bl/6 mouse lungs were conditioned with lysophosphatidylcholine, followed one-hour later by a LV vector carrying the *luciferase* reporter gene, using six different short-term (≤1 wk) and long-term (>1 wk) dosing schedules. Luciferase expression was quantified using bioluminescence imaging over 12 months. Most dosing schedules produced detectable bioluminescence over the 12-month period, but the shorter intervals (≤1 wk) produced higher levels of flux than the longest interval (five doses at least 1-month apart). Ex vivo lung analysis at 12 months showed that the estimated mean flux for the group that received two doses 1-week apart was significantly greater than the single dose group and the two groups that received doses over a period greater than 1-week. These results suggest that early consecutive multiple doses are more effective at improving gene expression in mouse lungs at 12 months, than longer repeat dosing intervals.

## Introduction

Cystic fibrosis (CF) is a recessive genetic disorder caused by pathogenic variants of the cystic fibrosis transmembrane conductance regulator (*CFTR*) gene. *CFTR* encodes for an epithelial chloride channel, and although CF affects multiple organ systems it is the progressive lung disease that is the major cause of mortality and morbidity. While the average life expectancy of individuals with CF has increased considerably to approximately 46 years of age, due to earlier diagnosis and significant advances in symptomatic therapies [1], quality of life continues to be impacted.

The introduction of CFTR modulators that act to correct and/or potentiate CFTR channel function has provided substantial clinical benefit to many individuals living with CF. However, not everyone with CF is eligible for these modulator therapies, with 10% of the CF population carrying rare CFTR variants left without an effective treatment [2]. There is also a high cost associated with these daily pharmaceuticals, with several national health systems declining to recommend government financial support to provide CFTR modulators [3]. Some CFTR modulators are also poorly tolerated by eligible people with CF, with approximately 23% either fully or temporarily discontinuing their treatment, due to a mix of respiratory and non-respiratory side effects [4]. Despite the continued development of CFTR modulators, the need for a mutation-agnostic treatment that will provide long-term therapeutic benefit remains.

Gene-addition therapy offers the potential of a curative universal treatment for CF lung disease by using a vector to deliver wild-type *CFTR* to the relevant airway cells to correct the underlying genetic defect for all CF mutations. A range of viral and non-viral gene vectors have been developed for CF [5]. The most widely researched are adeno-associated virus (AAV) vectors. AAV can be serotyped to target respiratory cells, with a range of new serotypes recently developed [6], and clinical-grade vector can be readily produced and purified at high titre. However, AAVs have limitations including a small packaging capacity that precludes the use of full-length *CFTR*, and they remain largely episomal, resulting in transient airway gene expression [7, 8]. Lentiviral (LV) vectors exhibit a range of factors that potentially make them a more appropriate option for clinical development. They have a large packaging capacity; they integrate the transgene into the genome of dividing and non-dividing cells, providing persistent gene expression in the transduced cells as well as their progeny [9]; they can be easily pseudotyped to alter their tropism for airway cells [10]; and they are considered safer than other viral vectors due to their low immunogenicity and toxicity [11]. However, LV vectors do have disadvantages including potential safety concerns associated with the risk of insertional mutagenesis.

Long-term reporter gene studies have demonstrated that a single LV vector dose results in persistent transgene expression in mouse airways [10, 12–14]. *CFTR* gene transfer studies in the nasal airways of CF mice have shown that LV vectors can correct CFTR function for up to 12 months following a single dose [15, 16]. In mice, *CFTR* expression eventually waned over time, and in some animals reduced to zero [16]. Although these studies validated the efficacy and durability of airway gene expression, a single-dose delivery may be insufficient to produce a lasting therapeutic benefit for the lifetime of a person with CF [14].

A significant translational challenge associated with all gene vectors is the ability of the vector to be redosed when initial transgene expression declines. The innate and adaptive immune responses to AAV [17] have been previously described. For AAV repeat dosing to be feasible, overcoming pre-existing AAV antibodies in people previously exposed to AAV and reducing neutralising antibody responses produced by multiple deliveries will be essential [18–20]. The immune responses to LV vectors are less clear [21]. The potential to repeatedly administer a F/HN pseudotyped LV vector derived from the simian immunodeficiency virus (SIV) to the nasal epithelium of mice has previously been examined [14]. Daily repeated administration for 10 days produced a dose-related increase in airway gene expression levels, suggesting early repeat dosing could be beneficial. In addition, two doses of a GFP reporter (F/HN-SIV-GFP) followed by a single luciferase reporter (F/HN-SIV-Lux) over a two-month period did not result in reduced *luciferase* gene expression compared to a single luciferase dose (F/HN-SIV-Lux), suggesting that exposure to the F/HN vector does not reduce the efficacy of later doses [13, 14]. Similarly, a study using a GP64-pseudotyped feline immunodeficiency virus (FIV) demonstrated that daily nasal dosing for seven days produced a linear increase in gene expression at the 12-week study endpoint [22]. Priming doses of GP64-FIV did not cause a loss of expression from a later dose, and may in fact have been beneficial. When a VSV-G pseudotyped FIV vector was used in that same study no expression was observed, likely because an airway conditioning agent that disrupts tight junctions was not used.

The aims of the present study were to examine whether a VSV-G pseudotyped HIV vector in conjunction with lysophosphatidylcholine airway conditioning could be successfully readministered to mouse lungs, and if repeated doses resulted in superior gene expression levels and longevity when compared to a single dose. We hypothesised that repeated LV gene vector administration would result in higher gene expression levels when compared to a single dose. In particular, we theorised that repeat dosing can be used to increase total gene expression through (1) multiple closely-spaced early doses to increase initial gene expression, and (2) repeat-dosing over longer periods to maintain gene expression.

## Materials and methods

**Fig. 1:**
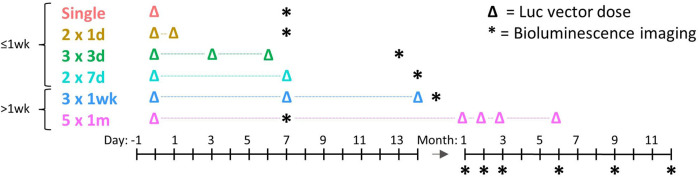
Study timeline. Note that the interval between any LV dosing event and bioluminescence imaging measurement was at least 1-week. Note that  means two doses one-day apart and  means three doses 1-week apart, etc.

This study was conducted under the approval of the University of Adelaide (M-2017-111) and Women’s and Children’s Hospital (AE1083) animal ethics committees, and in accordance with the ARRIVE guidelines [23]. The study used a LV vector containing the *luciferase* transgene to assess the impact of the dosing schedule on bioluminescence levels over a 12-month period (Fig. 1). The first four groups (≤1 wk) were designed to assess hypothesis one, with all dosing performed over a maximum of 1-week. The last two groups (>1 wk) were designed to assess hypothesis two with dosing performed over 14 days () and 6 months (). LV vector dosing, bioluminescence imaging (BLI) were performed as shown in the schedule. No mock treatment controls were used in this study because our previous studies have demonstrated that there is no auto-bioluminescence in the absence of LV-Luc delivery [16].

### Lentiviral vector production

VSV-G pseudotyped HIV-1 derived LV vector was produced using a previously described method [24]. The bicistronic LV-3xFLAG-Luc-GFP vector (denoted LV-Luc) expressed firefly *luciferase* (Luc) and *green fluorescent protein* (GFP) genes under the control of the EF1α promoter. To determine the functional titre HEK 293T cells were seeded onto 12-well plates, transduced, harvested, and fixed. Flow cytometric analysis was performed to determine the number of GFP-positive cells [25]. The final titre of the LV-Luc vector was 9.4 × 10^6^ TU/mL.

### Airway gene transfer

Airway gene transfer was performed as described in Cmielewski et. al (2017) [26]. For all LV-dosing procedures, female C57Bl/6 mice (8–10 weeks of age at study commencement) were anaesthetised with an i.p. injection of a 10 µl/g body weight of a mixture of medetomidine (0.1 mg/ml, Orion Corp., Finland) and ketamine (7.6 mg/ml, Parnell Laboratories, Australia). Anaesthetised mice were non-surgically intubated with a 20 Ga i.v. catheter (BD Insyte, Becton Dickinson, USA), placed on a heating mat in a supine position and observed prior to any fluid delivery to ensure breathing was normal. The airways were conditioned with 10 µl of 0.1% lysophosphatidylcholine (LPC; Sigma-Aldrich) and one-hour later, 20 µl of the LV vector was delivered into the trachea in two 10 µl aliquots delivered ~60 s apart. The endotracheal tube was then removed, and anaesthesia was reversed with 2 µl/g body weight i.p. injection of atipamezole (0.5 mg/ml, Orion Corp., Finland). Animals were dosed according to the assigned dosing schedules outlined in Fig. 1 (*n* = 12 mice randomly assigned to each group). Sample sizes were determined based on previous studies.

### Assessment of reporter gene expression

*Luciferase* gene expression was quantified using BLI (IVIS Lumina XRMS in vivo imaging system, PerkinElmer, Massachusetts, USA) at various imaging time points indicated in Fig. 1. Investigators were not blinded to the group allocation during the bioluminescence imaging. Mice were anaesthetised as described above and 50 µl of 15 mg/ml D-luciferin (Cayman Chemicals, US) was delivered to both nostrils as a bolus over 10 to 20 s. Ten minutes later each animal was imaged following a previously described method [10]. The resultant photon flux was determined in a region of interest created using the auto contour parameter measurement tool with background correction. After the final imaging time point, all mice were humanely killed with sodium pentobarbital (150–300 mg/kg i.p.) while under anaesthetic. Lungs with the trachea attached were immediately excised and re-imaged ex vivo in a small petri dish containing phosphate-buffered saline [10], to remove the obstructive effect of body tissues and fur on the detectable bioluminescence.

### Immunohistochemistry

Lung samples from untreated (negative control) and LV-treated mice were fixed in 4% paraformaldehyde, paraffin-embedded, sectioned at 5 µm, and de-paraffinised using standard histological procedures. Antigen retrieval was performed using 0.1 M EDTA (pH 8.0) for 10 min followed by permeabilisation in phosphate-buffered saline (PBS)/Triton X-100 (0.3%) for 10 min. Sections were blocked for 1 h at room temperature in a solution of 1% bovine serum albumin and 0.05% Tween-20. Primary antibody chicken α-GFP (1:100 dilution) (Abcam, ab13970) was resuspended in blocking buffer and incubated with samples overnight at 4 °C. No antibody and secondary antibody-only controls were included. Slides were washed followed by incubation with goat α-chicken Alexa Fluor® 568 (1:200) (Abcam, ab175711) in blocking buffer at room temperature for 1 h. Samples were washed followed by counterstaining with DAPI (1 µg/mL) and mounting with ProLong Diamond Antifade Mountant (Molecular Probes). Images were generated using an Olympus FV3000 confocal microscope.

### Statistical analyses

Statistical analysis was performed in a similar manner to that described for our previous examinations of luciferase bioluminescence [10]. Statistical significance was set at *p* = 0.05 and the flux limit of detection was set to 100.

To compare the effect of the different treatments on in vivo bioluminescence over time we fitted a linear mixed model to log-transformed flux values using the “lme” function from the “nlme” package [27] in R version 4.1.1 [28]. We fitted treatment, time (as a factor) and their interaction as fixed effects, and animal ID as a random effect, with an exponential correlation structure to account for within-animal autocorrelation over time (as a continuous variable). The within-subject variance was estimated separately for each treatment. Some mice had a flux value below detection but at a later time point returned a subsequent value that was above the detection limit. In those cases, the early values that were below the detection limit were set to missing. For the animals where lung bioluminescence did not return, those values were set to the limit of detection.

To look at the effect of the different treatments on flux in the ex vivo lung tissue excised after the animals were humanely killed, we fitted a one-way ANOVA to log(flux) after checking for normality. Tukey’s multiple comparisons were used to assess differences between groups.

## Results

Repeat administration of both LPC and the LV gene vector during all fluid delivery procedures, and the BLI assessments were well tolerated, and post-procedure weight was maintained as expected. A total of *n* = 66/72 (92%) survived all gene vector dosing procedures. However, three animals could not receive a LV dose due to problems with intubation and were therefore removed from the study. In the  group, two animals died under anaesthesia following the second LV dosing procedure, 24 h after the initial dose. One animal assigned to the  group was humanely killed, as it did not maintain its post-procedure weight following its first LV dose. One mouse in the  group died after the nine-month BLI due to respiratory failure while under injectable anaesthesia.

**Fig. 2:**
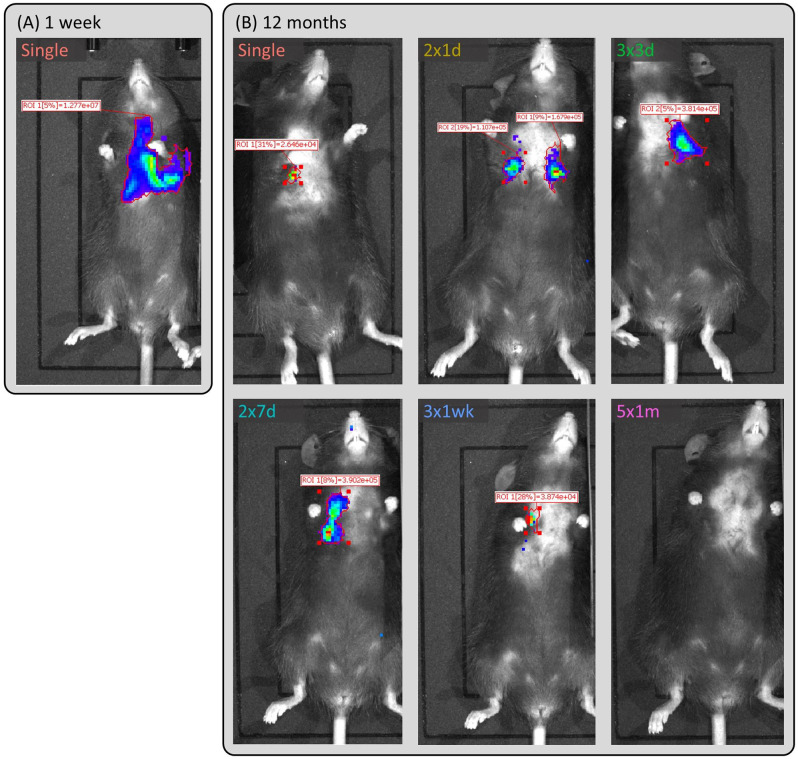
Example in vivo luciferase bioluminescence imaging results. (**A**) 1-week and (**B**) 12 months. The  dose animal is representative of all groups at 1-week. The 12-month animals shown here were chosen as they had a flux value that was closest to the group estimated mean flux from the fitted model.

**Fig. 3:**
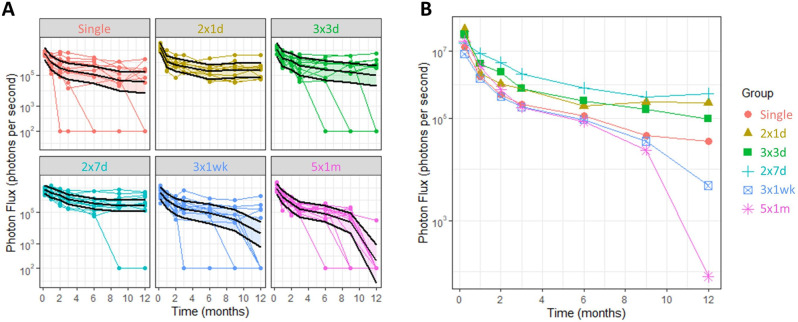
In vivo luciferase bioluminescence imaging results. (**A**) individual trajectories and estimated means and 95% CIs over the 12-month period, and (**B**) group means plot. *n* = 9–12 mice/group.

In vivo luciferase reporter gene expression (photon flux) was monitored non-invasively by BLI at multiple imaging time points over a 12-month period. Examples of the in vivo BLI measurements are shown in Fig. 2. In all groups other than the  group there were some animals for which no luminescence was detected at a particular time point, but for some animals luminescence returned at later time points (raw data shown in Supplementary Fig. 1). The model was applied as described in the methods, producing the fitted splines shown in Fig. 3.

### Comparison between dosing groups

All dosing schedules achieved high levels of luminescence at the first imaging time point, with no statistically significant differences measured between groups (Fig. 3B). The model was fitted to log(flux), so the pairwise comparisons were back-transformed to the original scale to calculate an estimated ratio of the means. There was an interaction between treatment and time (*F*_30,343_ = 3.59, *p* < 0.0001), which indicated that the level of luciferase expression over time changes depending on the dosing regimen used. We found that at nine months the estimated mean flux for the  group was significantly higher than the  group, and at 12 months the  group and  group were higher than the  wk group (Table 1). At 12 months the , ,  and  groups were all significantly higher than the  group.

**Table 1:** Significant differences in the estimated mean flux were found for some groups at the 9 and 12-month time points. The G1:G2 ratio indicates how much higher the flux was in Group 1 than Group 2.

### Comparison across time points

For all treatment groups, the level of lung bioluminescence at all imaging time points from 1-month to 12 months was significantly lower compared to the first imaging time point. The smallest reduction in estimated mean flux at 1 month compared to the first BLI measurement occurred in the  group (Table 2), and the largest reduction was in the  group. In the ,  and  groups, there were no significant reductions in flux at any of the latter time points compared to the 2-month time point, but in contrast, the ,  and  groups all had large and statistically significant reductions in flux over that period (data not shown). This effect was strongest in the  group, which had an estimated mean flux that was 123,000× lower at 12 months compared to the first time point, which contrasted with reductions of 465× for the  group and 44× for the  group (Table 2).

**Table 2:** Reductions in estimated mean flux over time, from 1-week to 1-month and 1-week to 12 months (other time points not shown). The T1:T2 ratio indicates flux at Time 1 is higher than at Time 2, with larger numbers indicating greater reductions.

### Ex vivo and immunohistochemical analyses

**Fig. 4:**
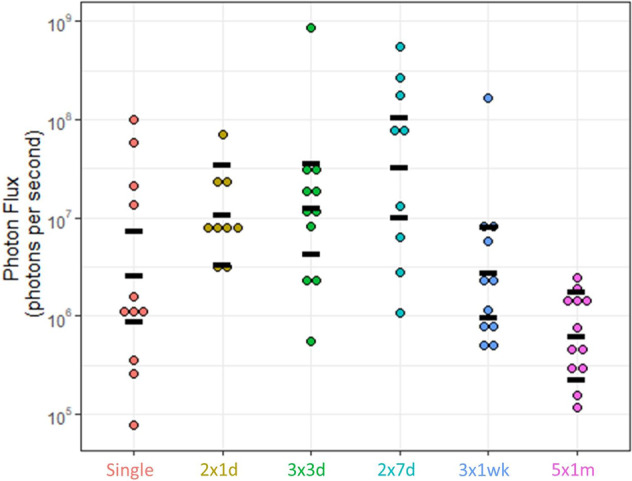
Ex vivo flux measurements for each of the dosing schedules. Black bars indicate the mean and 95% CI. *n* = 9–12 mice/group.

**Fig. 5:**
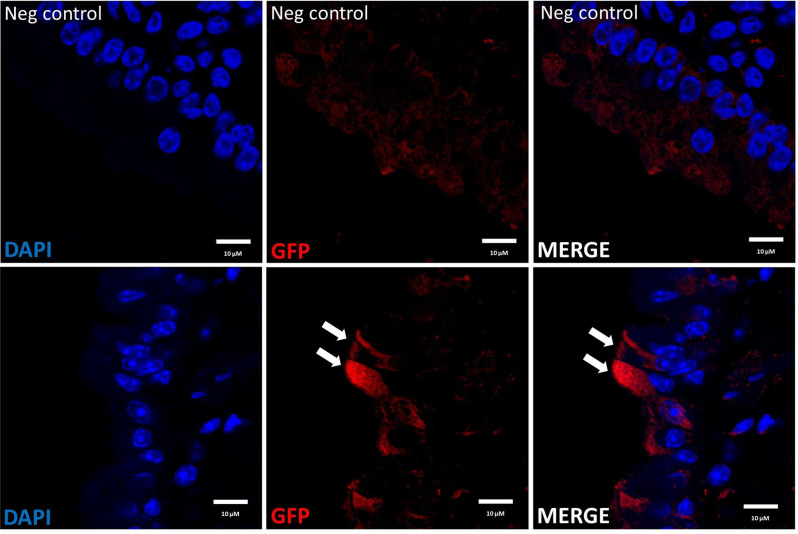
Detection of GFP positive cells in mouse airways via immunohistochemistry. Left: Nuclei are stained blue with DAPI, Middle: GFP positive cells are stained red, Right: Merged images. Top panel: Negative control mouse that did not receive LV vector treatment, Bottom panel: Airway from a mouse in the 2 × 7d group, showing surface epithelial cells expressing the GFP transgene.

Analysis of the ex vivo BLI data at the final 12-month imaging time point (Fig. 4) found there were significant differences between the groups (*F*_5,57_ = 6.85, *p* < 0.001). The estimated mean flux for the  group was higher than our standard  dose group, and higher than the  and  groups (Table 3). The  and  groups also had higher flux than the  group. GFP-positive cells could be detected in lung tissue at 12 months post-dosing, primarily in the conducting airways (Fig. 5).

**Table 3:** Estimated mean flux comparisons in the ex vivo tissues for each dosing schedule. The G1:G2 ratio indicates how much higher the flux was in Group 1 compared to Group 2.

## Discussion

Airway gene-addition therapy could offer lasting benefits for people with CF, particularly those that do not have access to highly effective CFTR modulator therapies. Amongst all the potential gene transfer vehicles, LV vectors have been demonstrated to have characteristics that make them particularly appealing, one of which is their low immunogenicity which makes redosing possible. The ability to increase levels of initial gene expression, or boost levels when they wane over time will likely be vital for any vector delivery system. Although LV redosing has been examined in the past [14, 22], neither the use of the VSV-G pseudotype nor the use of LPC conditioning in a repeat-dosing scenario have been thoroughly examined. The primary aim of this study was to determine the optimal dosing schedule of a VSV-G pseudotyped LV vector delivered into mouse lungs.

Our first hypothesis was that multiple closely-spaced early doses would increase initial lung gene expression levels, resulting in better long term expression when compared to a single dose. This was based on Griesenbach et al. who found evidence for the effectiveness of early multiple doses, reporting that five or ten daily doses of a F/HN-SIV-Lux vector both significantly increased nasal and lung luciferase expression compared to a single dose [14], and Sinn et al. who reported an increase in nasal luciferase expression following seven doses over a 1-week period [22]. In our study, there was no evidence from the in vivo flux assessments that any dosing schedule produced significantly higher mean expression than the  dose group at any imaging time point. However, in the ex vivo BLI assessment of lungs removed at the end of the study, the estimated mean flux for the  group was higher than our standard  dose group, providing some evidence that multiple early doses are advantageous. Interestingly, there was not a strong benefit from more than two doses. The period between those two doses appeared to be important, because the  group had the largest within-group reduction in flux (36.9×) at 1-month compared to the first imaging time point, while the  group had the smallest reduction (2.39×). Whether this timing makes a difference in a clinical context remains unknown.

We speculate that there could be an unintended consequence of using multiple LPC deliveries. It is feasible that some transgene-expressing cells are removed by subsequent LPC conditioning doses, which means that multiple LPC administrations might in fact be detrimental. In addition, regardless of the dosing regimen chosen, expression levels in all groups waned by at least one log by three months, potentially due to the turnover of terminally differentiated transduced cells [29]. Together this data suggests that further studies are needed to examine whether the benefit from multiple gene vector deliveries is outweighed by multiple LPC deliveries.

It is important to note that there was higher mortality in the  and  groups, due to the impact of the injectable anaesthetic and the greater challenges associated with LPC and LV delivery to the mouse lung compared to the nose. This likely limits the number of doses that can be delivered to mouse lungs at the early time points. An inhalable anaesthetic such as isoflurane could reduce this mortality, but its use in conjunction with airway conditioning can be complicated due to the one-hour interval between LPC conditioning and LV delivery required for optimal gene transduction. Finally, although BLI could not detect increased levels of luciferase expression over the 12-month period, the levels of flux we achieved in the lung in this study were orders of magnitude higher than those previously reported in the mouse nose and lung following multiple doses [14, 22]. Our choice of vector pseudotype and promoter could be responsible for these differences.

Our second hypothesis was that repeat-dosing over longer periods would help maintain gene expression over time, but our results suggest that using dosing periods of longer than seven days (e.g.  and ) was detrimental to expression levels, compared to the standard  dose group when measured in vivo and ex vivo. Our data was also in contrast to Sinn et al. who reported a dose-dependent increase in nasal luciferase expression following three, five, or seven 1× weekly doses [22]. Nonetheless, taken together, our in vivo and ex vivo data suggest that the  group produces the highest levels of flux of all the dosing groups. The immunohistochemical analysis demonstrated the presence of GFP-positive cells in the conducting airways, which supports the data from our previous LV-LacZ reporter gene studies [10, 26].

In this study, we performed a sensitivity analysis to ascertain how to handle the BLI data from the animals that had no detectable flux after dosing with LV-Luc. This analysis was necessary because some animals had no detectable flux at a particular time point, but the flux at subsequent time points had returned to previous levels. This could have been due to the non-uniform localisation of D-Luciferin in the lung (see below), which may have confounded the findings. One approach was leaving these values as being at the limit of detection (100) but because this value was two to three logs lower than the other values this skewed the data sets. An alternative approach involved treating all BLI values at the detection limit as missing values, but this meant that animals that really had no luciferase expression were inappropriately excluded. As a balance between these two options, we chose to only set values as missing if they returned to above the detection limit.

The strength of this study is that it was a well-powered longitudinal assessment of a range of LV-vector lung dosing strategies over a long period of time (12 months). However, the study had some limitations. Although our chosen statistical analysis approach dealt with the flux values at the limit of detection, their presence could have been due to physiological variability in the D-luciferin nasal dosing approach. There may have been mismatches between the locations within the lung that were targeted by the LPC conditioning and vector delivered via an endotracheal tube, and the delivery of D-luciferin via nasal sniffing. However, due to welfare concerns it was not possible to intubate each animal at every BLI time point for D-luciferin delivery. Other groups have delivered D-luciferin via i.p. injection [22], but in our experience this produces much lower flux values. We also speculate that the sensitivity of the IVIS system for detecting small increases in flux from the repeat dosing schedules is low. In future studies, it would be advantageous to assess whether circulating antibodies (and other immune/cytokine responses) correlate with the decline in expression levels, particularly for the 5 × 1 m dosing schedule that had the largest reduction in gene expression at 12 months. In addition, the effect of the presence of the GFP transgene in the LV-Luc vector is unknown, and it is also possible that immune responses may be different if a higher titre vector is used. Finally, while our results and those of Sinn et al. and Griesenbach et al. are biologically and mechanistically interesting reporter gene studies, further development and testing with therapeutic *CFTR* gene vectors is required [14, 22].

The conclusions from our study are that repeat-dosing a VSV-G pseudotyped LV vector to the murine lung is feasible, but that longer repeat-dosing intervals are detrimental to expression levels compared to a  dose. There was also some evidence to suggest that the short interval  group produced higher ex vivo flux than our standard  dose group. However, further detailed examination, potentially including the use of small non-human primate repeat-dosing feasibility studies, to model the responses of the human lung and immune system more accurately, is necessary for clinical development.

## Supplementary information

Supplementary Figure 1

## Data Availability

The data generated in this study is available from the authors on reasonable request.
