# Supplementary figures and images for: Repeat or single-dose lentiviral vector administration to mouse lungs? It’s all about the timing
Source: Gene Ther. 2023 May 10;30(9):698–705. doi: 10.1038/s41434-023-00403-3 (PMC10506910; doi:10.1038/s41434-023-00403-3)

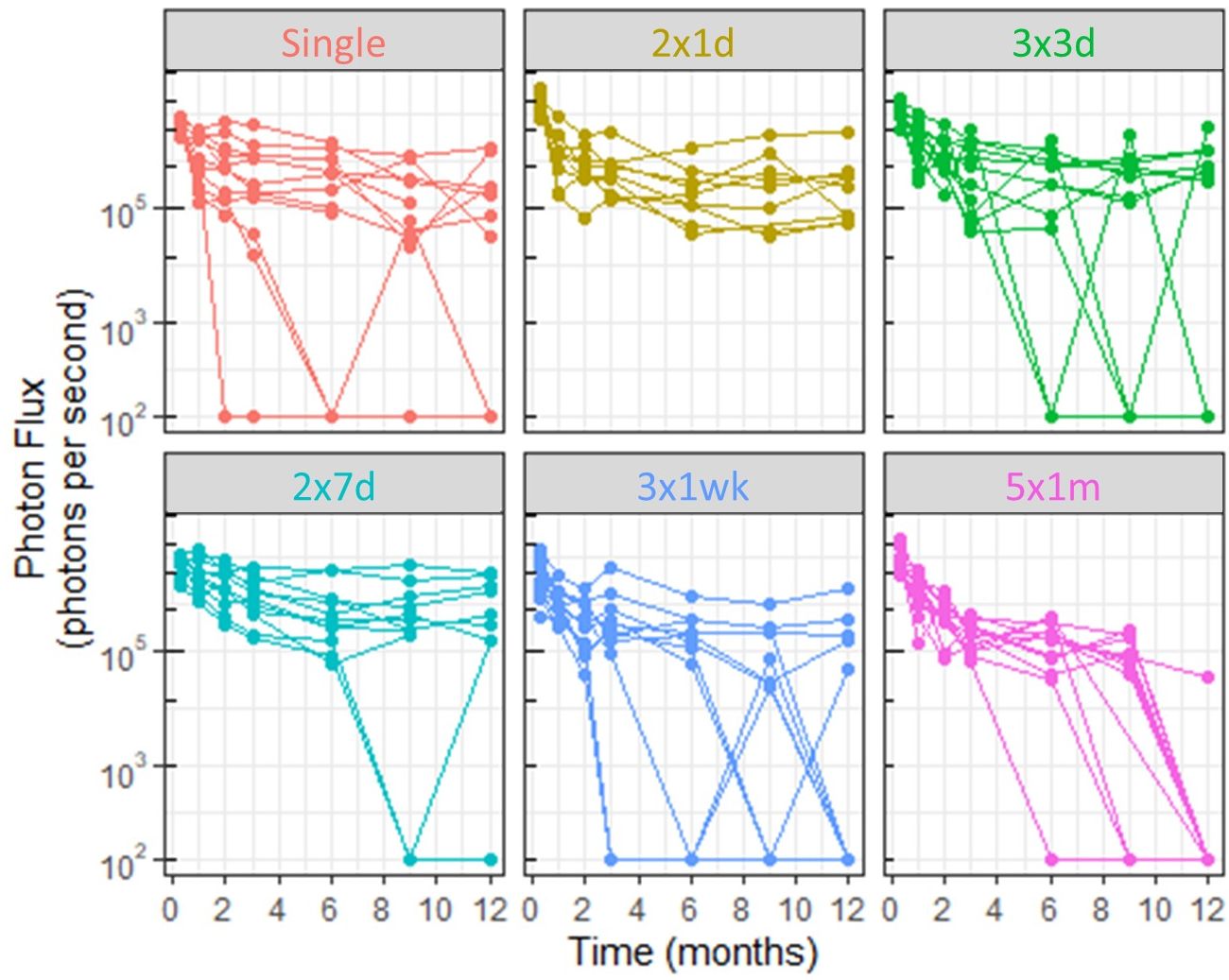

Supplement: Supplementary file 1 — Supplementary Figure 1 [file 41434_2023_403_MOESM1_ESM.jpg]
